# Weak and strong ties and its connection to experts' problem-solving styles in scaffolding students' PBL activities on social media

**DOI:** 10.12688/f1000research.73210.1

**Published:** 2021-10-25

**Authors:** Aznur Hajar Abdullah, Tse Kian Neo, Jing Hong Low

**Affiliations:** 1 Faculty of Management, Multimedia University, Cyberjaya, Selangor, Malaysia, 63100, Malaysia; 2 Centre for Adaptive Multimedia, Education and Learning Content Technologies (CAMELOT), Multimedia University, Cyberjaya, Selangor, 63100, Malaysia

**Keywords:** Problem-based learning, Facebook, business experts, problem-solving styles

## Abstract

**Background:** Studies have acknowledged that social media enables students to connect with and learn from experts from different ties available in the students’ personal learning environment (PLE). The inclusion of experts in formal learning activities through social media such as in scaffolding problem-solving activities helps students see the practicality of experts’ thinking in solving real-world problems. However, studies that evaluate experts’ problem-solving styles and how these influence the experts' thinking process in delivering the know-how to students on social media based on the ties that the students have with the experts in social media are scarce in the extant literature. The study aimed to explore the problem-solving styles that the experts portrayed on Facebook based on their ties with the students.

**Methods:** This study employed a simultaneous within-subject experimental design which was conducted in three closed Facebook groups with 12 final year management students, six business experts, and one instructor as the participants. The experts were invited by the students from the weak and strong ties in their PLE. Hinging on the Theory of Fluid and Crystallised intelligence and the Strength of Weak Ties Theory, this study employed thematic analysis using the ATLAS.ti qualitative data analysis software to map the experts’ comments on Facebook.

**Results:**  The use of strong ties in combination with weak ties balances out the negative aspects of the business experts’ problem-solving styles.  All the experts used both fluid and crystallised intelligence in scaffolding the students’ learning; however, the degree of its usage correlated with the working experience of the experts.

**Conclusion:** The use of weak or strong ties benefited the students as it expedited their problem-solving tasks since the experts have unique expertise to offer depending on the degree of their working experiences and the proximity of the students’ relationship with the experts.

## Introduction

### Background

Personal learning environment (PLE) is a self-driven learning space that allows individuals to collaborate, connect and participate using one or more technological artifacts, platforms, or online tools available in the personal learning space. ^1^ Siemen, ^2^ the founder of social connectivism theory, asserted that the inclusion of PLE is vital in online learning as students could form connections with external sources of more experienced people from dispersed geographical locations that could contribute knowledge and experiences that essentially aid students’ educational experience. ^3^

The use of social media embedded in students’ PLE enables students to gain access to experts who could support their formal and informal learning. ^4^^,^^5^ Social media allows students to tap into the connections of the weak ties from which they might draw resources. ^6^ In his famous strength of weak ties’ experiment, Granovetter ^7^ reported that people secure jobs mostly through weak ties by getting job information from acquaintances rather than close friends or family. Weak ties are defined by relationships that involve infrequent contact such as distant relatives, acquaintances, or people unknown to us. Meanwhile, strong ties refer to relationships of people who are closely in touch such as family members and close friends. Granovetter argued that although weak ties display low intimacy and emotional intensity than strong ties, it offers vital benefits such as providing more social support and networking strength. ^8^ It is reasonable to postulate that students could utilise their strong and weak ties by engaging with experts in their PLE on social media to support learning. Unfortunately, existing studies have not ascertained this assumption.

Recently, the use of experts to facilitate students’ learning in online settings has gained substantial attention among problem-based learning (PBL) scholars, mainly because expert thinking differs vastly from novice thinking. ^9^ Horn and Cattel‘s ^10^ Theory of Fluid and Crystallized Intelligence described experts as having more crystallised intelligence embedded than novices when dealing in knowledge-rich problem situations where the goals of a problem are uncertain and the solutions are not straightforward. ^10^^–^^12^

Therefore, experts devise solutions faster than novices because they use necessary knowledge based on their life experiences that are stored in long-term memory which makes up their crystallised intelligence. Additionally, experts also demonstrate fluid intelligence, namely the ability to reason and adapt without the need for substantial levels of prior learning when confronted with new problems or situations. This enables business experts, for instance, to accustom themselves to an ever-changing contemporary business environment characterised by volatility, uncertainty, fuzziness, and complexity. ^13^

In contrast, novices tend to lose direction when dealing with complex problem-solving, especially when confronting information that is presented simultaneously in an online context. Consequently, when placed in online platforms to solve complex problems, students often need a more experienced individual to guide their thinking to approximate the experts’ reasoning ^14^ and to reconcile the misunderstanding. The use of PBL in technology-rich environments such as social media allows students to receive online scaffolding, a form of assistance from more experienced people who could guide them in performing unfamiliar tasks they are incapable of performing on their own in online mediated platforms. ^4^ Students may integrate their PLE with unlimited arrays of scaffolders who are socially connected in social media including instructors, peers and experts to assist in the problem-solving tasks.

Several studies have investigated how experts deal with novices in problem-solving activities. ^15^^–^^17^ Nevertheless, very few have explored the patterns of experts’ problem-solving styles that are drawn via the use of strong and weak ties to support problem-solving activities with students.

Objectives and rationales

This study explored the patterns of experts’ problem-solving styles and intelligence characteristics when reasoning with students in problem-solving activities whereby the patterns were mapped against the ties the students established in their PLE. Since experts think differently from novices, understanding these patterns would help novices and educators gain insight into the scaffolding provided by experts.

## Methods

The sampling techniques and the instruments used were reported according to STROBE (Strengthening the Reporting of Observational Studies in Epidemiology) reporting guideline, ^18^ a popular guideline in social science research.

### Ethical approval and consent

This study was approved by the Research Ethical Committee of Multimedia University (EA2012021). Initially, all participants were briefed on the assignment deadlines and expected roles in the problem-solving protocols. Subsequently, written informed consent for participation and publication of the research has been obtained from the participants. All communications on Facebook were transcribed and their identities were concealed for maintaining the participants’ anonymity following STROBE guideline and Subirats *et al*. ^19^

Study design, setting and participants

The researchers made a call for volunteers who were undertaking a global management course at a Malaysian private university to participate in solving a decision-making business problem. The volunteers were required to invite along two business experts from their PLE to scaffolded them for eight weeks. The requirements of the business experts were set as follows: having substantial working experience of 10 years or more, hold a managerial position and the experts must have one of the following ties with the students; both experts are from strong, weak or both ties. Finally, 12 final-year baccalaureate students who met the research criteria volunteered to participate. This study used a simultaneous within-subject experimental design for three groups comprising four students each (two from Cohort 2017 and one from Cohort 2018) were assigned in a closed group Facebook to communicate, clarify issues, and share resources. Furthermore, this group arrangement is common in PBL studies. ^20^ Facebook was selected because of its effectiveness in supporting various degrees of ties and capability to accommodate small PBL groups. ^21^ The Facebook communications were transcribed and available in a dataset. ^22^ Students were scaffolded by the experts and instructor following Ge and Land’s ^23^ problem-solving protocol which involved problem identification, developing and evaluating solutions, and assessing alternative solutions.

The students documented their work on Google documents that could be assessed only by the instructor, experts and students for each respective group.

Table 1 depicts the business experts’ profiles. Groups 1 and 2 used weak ties. A student in Group 1 invited two experts from her former internship company during her diploma studies. Group 2 invited two experts whom the students searched from an organisation’s website; none of the students knew the experts before inviting them to participate in this study. Group 3 used a combination of weak and strong ties. The strong tie was one of the students’ close relatives while the weak tie was one of the student’s internship acquaintances. The business experts from Groups 1 and 3 have 20 to 30 years of work experience in the shipping and airport management industry, respectively. Meanwhile, the experts in Group 2 have 10-15 years of work experience in the e-commerce industry.

**Table 1.** The business experts’ profile.

Group	Ties	The industry that the business experts were engaged in and the assigned case.
1	Weak	Shipping industry https://www.nst.com.my/news/2016/03/132323/revival-hope-floats-shipping-master-plan
2	Weak	E-commerce industry https://www.digitalnewsasia.com/digital-economy/slow-internet-speeds-damping-malaysias-digital-economy-aspirations-mdec-ceo
3	Strong + Weak	Airport management http://www.thenational.ae/business/aviation/mattala-rajapaksa-airport-fails-to-take-off-as-sri-lankas-newest-destination

Methods of analysis

Friese *et al*. ^24^ recommended the use of deductive thematic analysis when a pre-existing framework is available. Therefore, the discussions between the business experts and the students were thematically mapped using Selby's ^25^ three problem-solving styles. These included problem-solving preferences (explorer vs. developer) which were coded as M: Explorer and M: Developer; the manner of processing (internal vs. external) coded as MP: Internal and MP: External; and finally, ways of deciding (people vs task preference) coded as WOD: People and WOD: Task. The Theory of Fluid and Crystallised Intelligence ^10^ was used to map the type of intelligence the experts primarily demonstrated. ATLAS.ti software (Version 8.4.25.0) was used to analyse the identified themes to reflect the business experts’ responses. Acknowledging that there is available open-source software as alternatives to ATLAS.ti such as QualCoder and Tagguete, many qualitative scholarly papers adopted ATLAS.ti for its user-friendliness for coding and displaying network analysis results. Besides that, ATLAS.ti has a variety of tools to analyse unstructured data. ^26^ Moreover, one of the researchers in this study is well-versed in using ATLAS.ti. For those reasons, ATLAS.ti was chosen.

## Results

**Figure 1.**
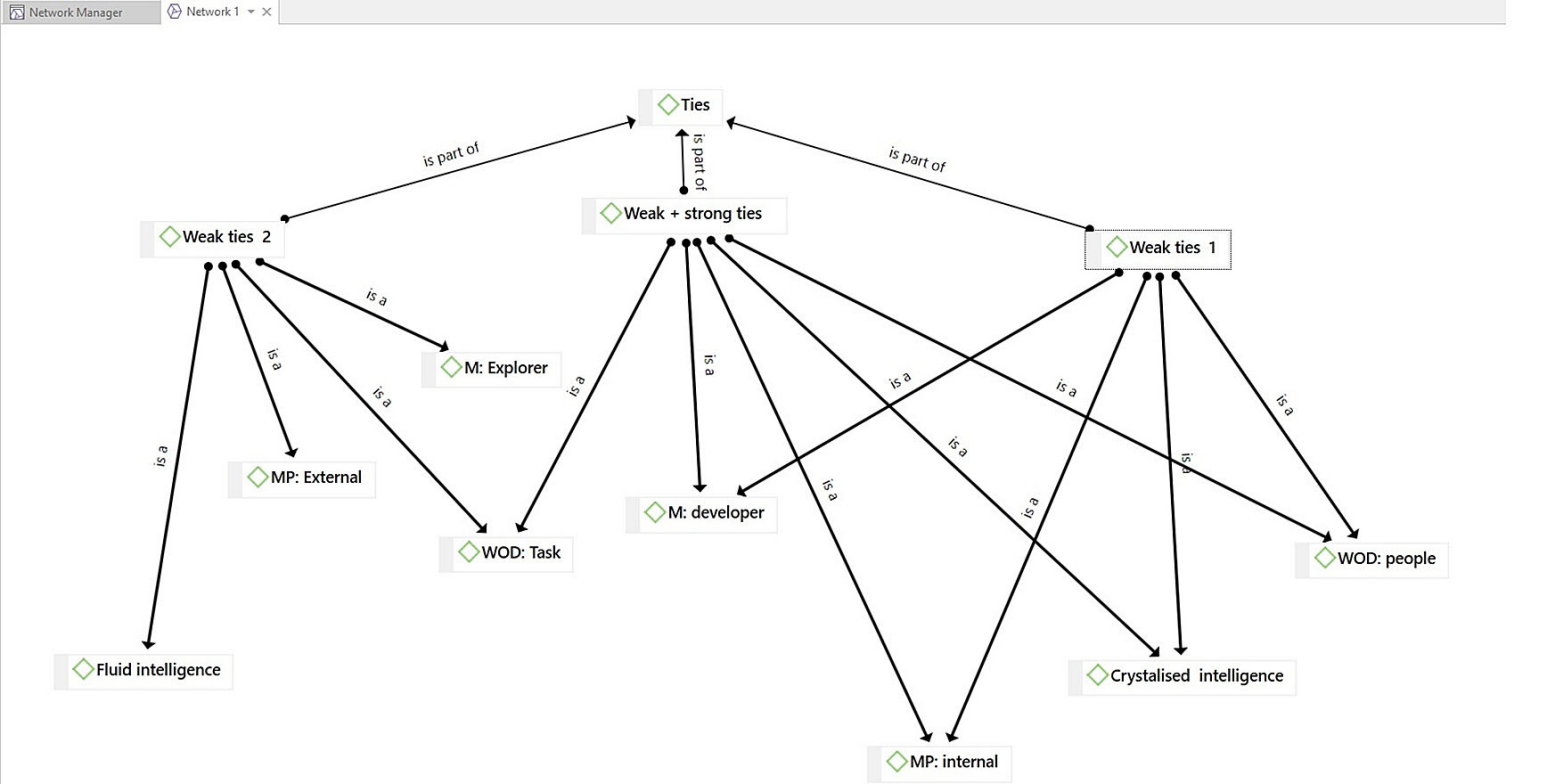
The Network Analysis from ATLAS.ti.

**Figure 2.**
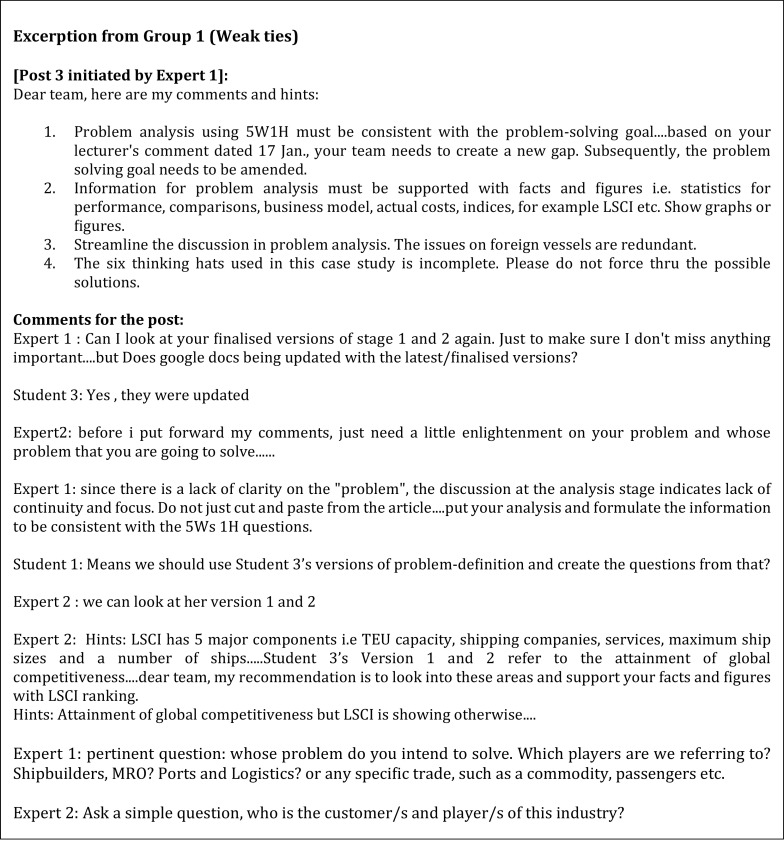
The weak tie from Group 1.

**Figure 3.**
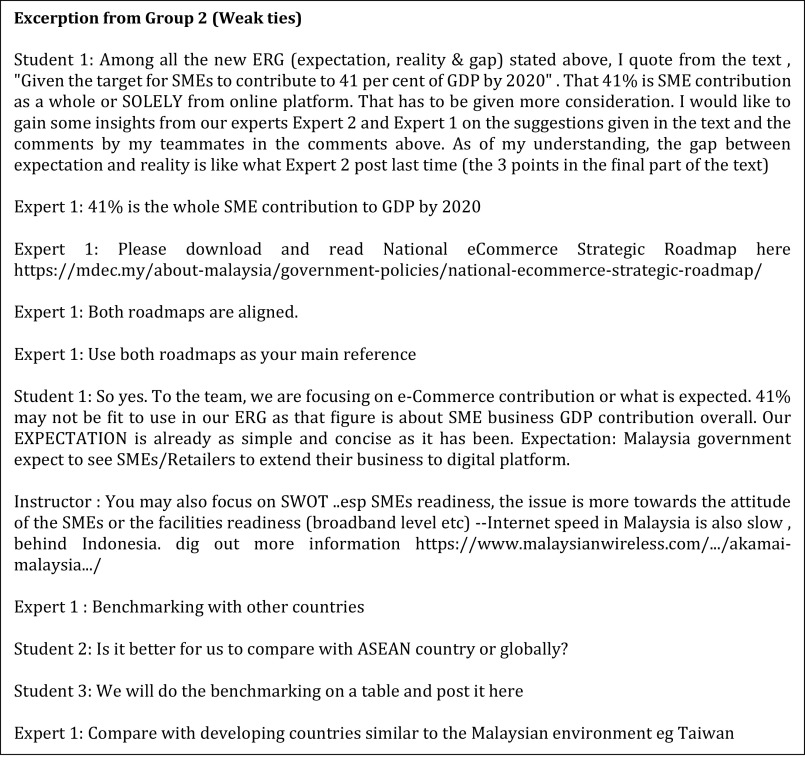
The weak ties from Group 2.

Figure 1 displays the network analysis based on the themes extracted from Facebook discussions. The weak tie experts in Group 1 ( Figure 2) displayed a more accommodating approach and a sense of belongingness by using phrases such as “dear team” and “keep moving team”. Selby ^25^ described this as the people preference style where this approach is seen as an effort to maintain harmony in the group. The experts respected the students' own pace of processing information as they required time to digest and internalise the meaning of the information presented to them by the students before responding. In contrast, the business experts from Group 2 (weak ties 2) adjusted their reasoning based on the information the students presented to them first. This sort of arrangement falls under the explorer style. The experts preferred the students exploring all possible options and presenting the latest information before guiding the students based on the materials presented ( Figure 3).

**Figure 4.**
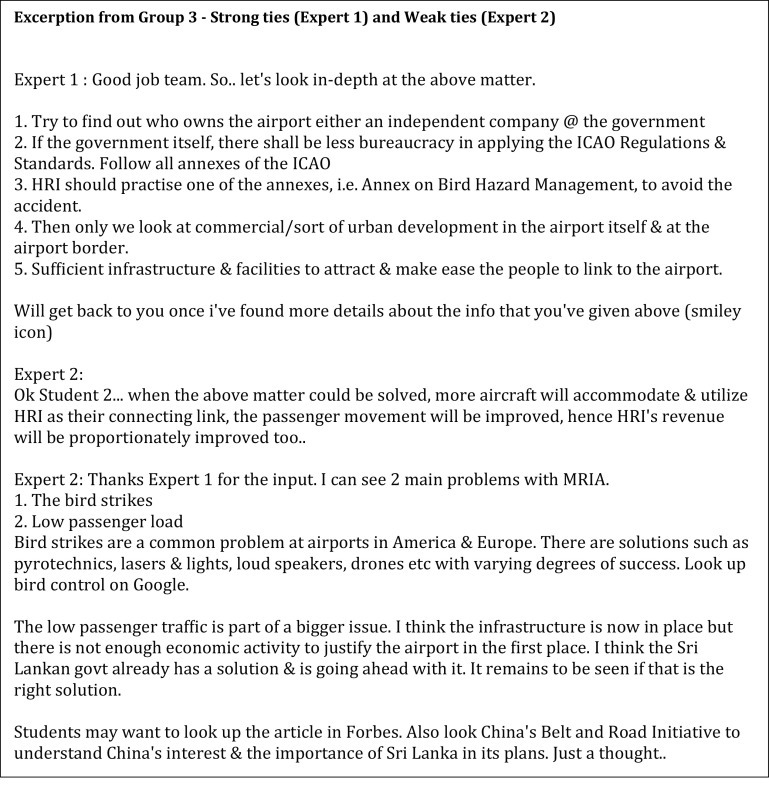
The strong and weak ties from Group 3.

Lastly, Group 3 which used a combination of both weak and strong ties ( Figure 4) showed mixed findings. The strong tie expert (Expert 1 from strong tie) was sensitive to the participants’ feelings and ended her comments with remarks such as “Otherwise, good job all”. This style is categorised as the people preference. The strong tie expert also demonstrated more persistence and patience in scaffolding the students by presenting the developer style. She directed the students beginning with a basic idea and gradually developed the ideas as the students were progressing by making statements such as “I think it would be a good idea if … .”. This characteristic is similar to the style of experts in Group 1. In contrast, the weak tie expert (Industry Expert 2) exhibited a task preference style where the tone of the discussion was more of task accomplishment tend to be free from emotion and are focused on the tasks, which sometimes resemble the explorer style ^25^ as the experts were inclined to share only after receiving information from the students.

These sorts of problem-solving styles of the experts correlated with the types of intelligence used and working experiences. Experts from Group 2 (with 10-15 years of work experience) exhibited more fluid intelligence as they were flexible in dealing with new information in thinking and reasoning with the students. In contrast, the business experts in Groups 1 and 3 (with more than 30 years of work experience) demonstrated crystallised intelligence and shared validated business solutions by occasionally sharing how the presented information was linked to their past experiences. The explanations given by the experts in Groups 1 and 3 were also seen as more insightful compared to the guidance provided by experts in Group 2.

## Discussion and conclusion

According to Bilalic and Gobet, ^17^ the greater the degree of expertise, the more flexible the experts are in responding to new information. The profile of the business experts from Groups 1 and 3 showed they have vast experiential knowledge, rendering them capable of deciphering information from different perspectives. The experts used more technical terms and jargons which necessitated the students to ask a second party to provide the meaning-making for them. Occasionally, the students were observed needing to rely on the other expert or instructor for the meaning-making process (to put the meaning in a context understandable to the students). This is supported by Ryberg ^20^ who claimed that placing students in different degrees of ties sometimes require different participants like the instructor to provide the interpretation of meaning.

Daniel Kahneman, ^27^ in his book “Thinking Fast and Slow”, outlined two thinking systems called System 1 and System 2. System 1 is fast and energy-efficient because it follows the “rule of thumb” and does not involve processing of details; as a result, System 1 thinking is full of shortcomings and biases. In contrast, in System 2, information processing is intricate, time-consuming, and expends more energy, especially when dealing with ill-structured problems. This is where the roles of experts could help in expediting students’ effort by simplifying the need to understand. For novice learners, using System 2 may require a longer time for information processing. Nonetheless, the availability of experts with more work experiences could shorten students’ thinking process because of the experiential knowledge the experts have that resembles their crystallised intelligence. This is consistent with previous studies that confirmed people tend to use more crystallised intelligence as they increase in age. ^10^

Additionally, the business experts who used more fluid intelligence had different reasoning styles with the students. Instead of offering the information asked by the students straightaway, the experts from Group 2 often asked the students to search for the materials first, and later worked on the materials together with the students. This was possibly done to avoid offering inaccurate advice as a result of using System 1 thinking. The experts needed to verify the information before formulating relevant strategies to scaffold the students. The experts from Group 2 mostly provided policy papers rather than offering specific real-life business evidence that the students could use as a reference. Possibly, the experts expected the students to put in the effort to search for the information first.

This study also verified that scholars should not equate all weak tie experts as sharing similar problem-solving styles. It is postulated that how the students knew the business experts matters. The business experts from Groups 1 and 3 were from the weak ties; however, the past working relationship that one of the students in each group had with the experts during internship placement led the business experts to display a more empathic attitude towards the students’ learning needs. In contrast, the business experts from Group 2 had no prior relationship with the students, thus their preference for using more task-oriented problem-solving styles. Nonetheless, despite their different styles, the inclusion of the experts in the discussion still accelerated the students' learning, in tandem with previous studies that acknowledged business experts’ inclusion in PBL enhances students’ learning experience. ^28^^,^^29^

## Conclusion

This study contributes towards our understanding of the roles of problem-solving styles and the strength of ties in problem-solving activities on Facebook. The use of networked learning in PBL depends on individualised networking and social collaboration that encourage content generation in problem-solving. ^21^ It can be concluded from the findings that not all experts from the weak ties have similar problem-solving styles. Factors such as the experts’ work experience and how the weak ties were developed played a major role in determining the experts’ problem-solving styles, which indirectly influenced their thinking and reasoning strategies with the students.

The experts, regardless of whether they were from weak or strong ties, still benefited the students in expediting their problem-solving tasks. Thus, inviting business experts to participate in formal learning on social media by utilising the strong and weak ties the students have should be encouraged as each expert has unique expertise to offer, especially in helping the students see the different sides of complex information that are essential to prepare them for future professional career.

### Limitations

The use of non-probability sampling involving two experts in each of the three groups in one degree-level management course limits the generalisability of the findings to other courses. Hence, the study’s findings should be evaluated with caution and may only be applied to similar studies, for example, those that examine Facebook use for PBL in management courses.

## Data availability

### Underlying data

Figshare: Facebook Discussions captured in ATLAS.ti, https://doi.org/10.6084/m9.figshare.16811542.

This project contains the following underlying data:

Datafile 1: Transcribed conversation of Group 1

Datafile 2: Transcribed conversation of Group 2

Datafile 3: Transcribed conversation of Group 3

Data are available under the terms of the Creative Commons Attribution 4.0 International license (CC-BY 4.0).
