# Weak and strong ties and its connection to experts' problem-solving styles in scaffolding students' PBL activities on social media

**DOI:** 10.12688/f1000research.73210.2

**Published:** 2021-12-16

**Authors:** Aznur Hajar Abdullah, Tse Kian Neo, Jing Hong Low

**Affiliations:** 1 Faculty of Management, Multimedia University, Cyberjaya, Selangor, Malaysia, 63100, Malaysia; 2 Centre for Adaptive Multimedia, Education and Learning Content Technologies (CAMELOT), Multimedia University, Cyberjaya, Selangor, 63100, Malaysia

**Keywords:** Problem-based learning, Facebook, business experts, problem-solving styles

## Abstract

**Background:** Studies have acknowledged that social media enables students to connect with and learn from experts from different ties available in the students’ personal learning environment (PLE). Incorporating experts into formal learning activities such as scaffolding problem-solving tasks through social media, allows students to understand how experts solve real-world problems. However, studies that evaluate experts’ problem-solving styles on social media in relation to the tie strength of the experts with the students are scarce in the extant literature. This study aimed to explore the problem-solving styles that the experts portrayed based on their ties with the students in problem-based learning (PBL) on Facebook.

**Methods:** This study employed a simultaneous within-subject experimental design which was conducted in three closed Facebook groups with 12 final year management students, six business experts, and one instructor as the participants. The experts were invited by the students from the weak and strong ties in their PLE. Hinging on the Strength of Weak Ties Theory (Granovetter, 1973) and problem-solving styles (Selby et al., 2004), this study employed thematic analysis using the ATLAS.ti qualitative data analysis software to map the experts’ comments on Facebook.

**Results:**  The experts from strong and weak ties who had a prior relationship with the students showed people preference style by being more sensitive to the students' learning needs and demonstrating firmer scaffolding compared to the weak ties' experts who had no prior relationship with the students. Regardless of the types of ties, all experts applied all manner of processing information and orientation to change but the degree of its applications are correlated with the working experience of the experts.

**Conclusion:** The use of weak or strong ties benefited the students as it expedited their problem-solving tasks since the experts have unique expertise to offer depending on the problem-solving styles that they exhibited.

## Introduction

### Background

The use of experts to facilitate students’ learning in online settings has gained substantial attention among problem-based learning (PBL) scholars, mainly because expert thinking differs vastly from novice thinking. ^1^ Novices tend to lose direction when dealing with complex problem-solving, especially when confronting information that is presented simultaneously in an online context. Consequently, when placed in online platforms to solve complex problems, students often need a more experienced individual to guide their thinking to approximate the experts’ reasoning ^2^ and to reconcile the misunderstanding. The use of PBL in technology-rich environments such as social media allows students to receive online scaffolding, a form of assistance from more experienced people who could guide them in performing unfamiliar tasks they are incapable of performing on their own in online mediated platforms. ^3^ Students may integrate their personal learning environment with unlimited arrays of scaffolders who are socially connected in social media including instructors, peers, and experts to assist in the problem-solving tasks. 

Personal learning environment (PLE) is a self-driven learning space that allows individuals to collaborate, connect and participate using one or more technological artifacts, platforms, or online tools available in the personal learning space. ^4^ Siemens, ^5^ the founder of social connectivism theory, asserted that the inclusion of PLE is vital in online learning as students could form connections with external sources of more experienced people from dispersed geographical locations that could contribute knowledge and experiences that essentially aid students’ educational experience.

The use of social media embedded in students’ PLE enables students to gain access to experts who could support their formal and informal learning. ^2^^,^^6^ Social media allows students to tap into the connections of the weak ties from which they might draw resources. ^7^ In his famous strength of weak ties experiment, Granovetter ^8^ reported that people secure jobs mostly through weak ties by getting job information from acquaintances rather than close friends or family. Weak ties are defined by relationships that involve infrequent contacts such as distant relatives, acquaintances, or people unknown to us. Meanwhile, strong ties refer to relationships of people who are closely in touch such as family members and close friends. Granovetter argued that although weak ties display low intimacy and emotional intensity than strong ties, it offers vital benefits such as providing more social support and networking strength. ^9^ Past studies also espoused weak ties provide better connection and support than strong ties. ^10^^,^^11^ The main reason is strong ties usually offer redundant and homogenous resources, which reduces the need to communicate. ^11^ Therefore, experts devise solutions faster than novices because they use necessary knowledge based on their life experiences that are stored in long-term memory which makes up their crystallised intelligence. Additionally, experts also demonstrate fluid intelligence, namely the ability to reason and adapt without the need for substantial levels of prior learning when confronted with new problems or situations. This enables business experts, for instance, to accustom themselves to an ever-changing contemporary business environment characterised by volatility, uncertainty, fuzziness, and complexity. ^13^

In contrast, novices tend to lose direction when dealing with complex problem-solving, especially when confronting information that is presented simultaneously in an online context. Consequently, when placed in online platforms to solve complex problems, students often need a more experienced individual to guide their thinking to approximate the experts’ reasoning ^14^ and to reconcile the misunderstanding. The use of PBL in technology-rich environments such as social media allows students to receive online scaffolding, a form of assistance from more experienced people who could guide them in performing unfamiliar tasks they are incapable of performing on their own in online mediated platforms. ^4^ Students may integrate their PLE with unlimited arrays of scaffolders who are socially connected in social media including instructors, peers and experts to assist in the problem-solving tasks.

Several studies have investigated how experts deal with novices in problem-solving activities, ^1^^,^^16^^–^^18^ very few have explored the patterns of experts’ problem-solving styles that are drawn via the use of strong and weak ties to support problem-solving activities with the students. Since problem-solving styles resemble an attitudinal dimension of individual personality ^15^ and are relatively stable over time, ^14^ understanding the styles of the experts in different learning settings is vital in developing meaningful learning opportunities with the students.

Objectives and rationales

This study explored the patterns of experts’ problem-solving styles when reasoning with students in problem-solving activities, whereby the patterns were mapped against the ties that the students established in their PLE. Since experts think differently from novices, understanding these patterns would help novices and educators gain insight into the scaffolding provided by experts from different ties.

## Methods

The sampling techniques and the instruments used were reported according to STROBE (Strengthening the Reporting of Observational Studies in Epidemiology) reporting guideline, ^19^ a popular guideline in social science research.

### Ethical approval and consent

This study was approved by the Research Ethical Committee of Multimedia University (EA2012021). Initially, all participants were briefed on the assignment deadlines and expected roles in the problem-solving protocols. Subsequently, written informed consent for participation and publication of the research has been obtained from the participants. All communications on Facebook were transcribed and their identities were concealed for maintaining the participants’ anonymity following STROBE guideline and Subirats *et al*. ^20^ Before conducting the study, all the participants were informed to set their Facebook accounts to a private setting and were assigned to a private and closed group Facebook to communicate, clarify issues, and share resources.

Study design, setting and participants

The researchers made a call for volunteers who were undertaking a global management course at a Malaysian private university to participate in solving a decision-making business problem. The volunteers were required to invite along two business experts from their PLE to scaffold them for eight weeks. In line with the objective of the study to evaluate the problem-solving styles of the experts that the students have in their PLE, allowing students to select experts from their own PLE is deemed appropriate. This coincides with Dabbagh and Castaneda ^4^ recommendation to encourage students to use their PLE in formal courses as a means to enrich students’ learning experience through interactions among the students, instructors and experts.

The requirements of the business experts were set as follows: having substantial working experience of 10 years or more, holding a managerial position and the experts have one of the following ties with the students; both experts are from strong, weak or both ties. Finally, 12 final-year baccalaureate students (aged 21 to 22 years old) from the Bachelor of Business Administration programme in a global management course that met the research criteria volunteered to participate in a simultaneous within-subject experimental design. Three groups, comprising four students each (two from Cohort 2017 and one from Cohort 2018) was assigned in a closed group Facebook to communicate, clarify issues, and share resources. Furthermore, this group arrangement is common in PBL studies. ^21^ Facebook was selected because of its effectiveness in supporting various degrees of ties and capability to accommodate small PBL groups. ^22^

Meanwhile, Table 1 depicts the business experts’ profiles. Groups 1 and 2 used weak ties. A student in Group 1 invited two experts from her former internship company during her diploma studies. Group 2 invited two experts whom the students searched from an organisation’s website; none of the students knew the experts before inviting them to participate in this study. Group 3 used a combination of weak and strong ties. The strong tie was one of the students’ close relatives while the weak tie was one of the student’s internship acquaintances. The business experts from Groups 1 and 3 have 20 to 30 years of work experience in the shipping and airport management industry, respectively. Meanwhile, the experts in Group 2 have 10-15 years of work experience in the e-commerce industry.

**Table 1.** The business experts’ profile.

Group	Ties	The industry that the business experts were engaged in and the assigned case.
1	Weak	Shipping industry https://www.nst.com.my/news/2016/03/132323/revival-hope-floats-shipping-master-plan
2	Weak	E-commerce industry https://www.digitalnewsasia.com/digital-economy/slow-internet-speeds-damping-malaysias-digital-economy-aspirations-mdec-ceo
3	Strong + Weak	Airport management https://www.thenational.ae/business/aviation/mattala-rajapaksa-airport-fails-to-take-off-as-sri-lankas-newest-destination

In order to monitor the progress, the students documented their work on a Google document and the link was pinned on Facebook that could be assessed only by the instructor, experts and students for each respective group. To optimally guide Facebook problem-solving discussions, this study followed Optima 7 Jump (e-learning) protocol of Rienties *et al*. ^23^ that is commonly used in business education, in the following sequential orders - (1) identifying difficult terms or concepts, (2) identifying the problem and its requirements (the goal of the problem), (3) gathering relevant information such as personal experiences, literature, a news report that is aligned with the learning goal, (4) presenting on the findings in the previous step, (5) discussing the answers to reach an agreement, (6) assessing learning goals if they are answered and (7) summarizing the key points of the entire discussion. We redesigned the Optima model by incorporating, Ge and Land’s ^24^ problem-solving protocol which involved problem identification, developing and evaluating solutions, and assessing alternative solutions to make the discussions and progression more structured. These protocols were briefed to all participants before they began the problem-solving. 

Methods of analysis

Friese *et al*. ^25^ recommended the use of deductive thematic analysis when a pre-existing framework is available. Therefore, the discussions between the business experts and the students were thematically mapped using Selby’s *et al.* ^15^ three problem-solving styles or known as VIEW: An Assessment of Problem-Solving Style.

Orientation to change identifies individuals’ preferences when dealing with new problems. This preference covers the cognitive dimension of problem-solving styles that are divided into explorer or a developer. Explorers enjoy initiating a broad range of tasks in a non-directional manner and thriving to see new possibilities and patterns emerge from the new information. Meanwhile, developers prefer the structuredness of the tasks and plans. They usually begin with the basic elements of a problem, then organise and build more complete, functional, and useful outcomes. Whereas, manners in processing information refers to the way individuals arrange information and its flow that can be identified as internal and external processing style. Individuals that prefer internal processing styles need more time to decipher all relevant information before sharing it with others. In contrast, individuals with an external processing style appear to be full of energy when engaging with others while seeking the inputs and expect others to do the same. They also revise ideas along the way.

Finally, ways of deciding are related to individuals’ preferences when deciding about options. They can be categorised as people preference or task preference. Individuals with people preference are more concerned with people’s feelings and emotions. They also show effort in maintaining harmony and positive relationship with other team members. Oppositely, individuals who are task preference are concerned with task accomplishment, seeking logical arguments to arrive at the most practical solutions, tend to be natural and act free from emotion.

The Facebook communications were transcribed and available in a dataset ^26^ ATLAS.ti software (Version 8.4.25.0) was used to analyse the identified themes to reflect the business experts’ responses. Acknowledging that there is available open-source software as alternatives to ATLAS.ti such as QualCoder and Tagguete, many qualitative scholarly papers adopted ATLAS.ti for its user-friendliness for coding and displaying network analysis results. Besides that, ATLAS.ti has a variety of tools to analyse unstructured data. ^27^ Moreover, one of the researchers in this study is well-versed in using ATLAS.ti. For those reasons, ATLAS.ti was chosen. Subsequently, the problem-solving preferences (explorer vs. developer) were coded as follows: orientation to change (OC) (OC: Explorer and PS: Developer), manners in processing information (MP) (MP: Internal and MP: External); and ways of deciding (WOD) (WOD: People and WOD: Task).

## Results

**Figure 1.**
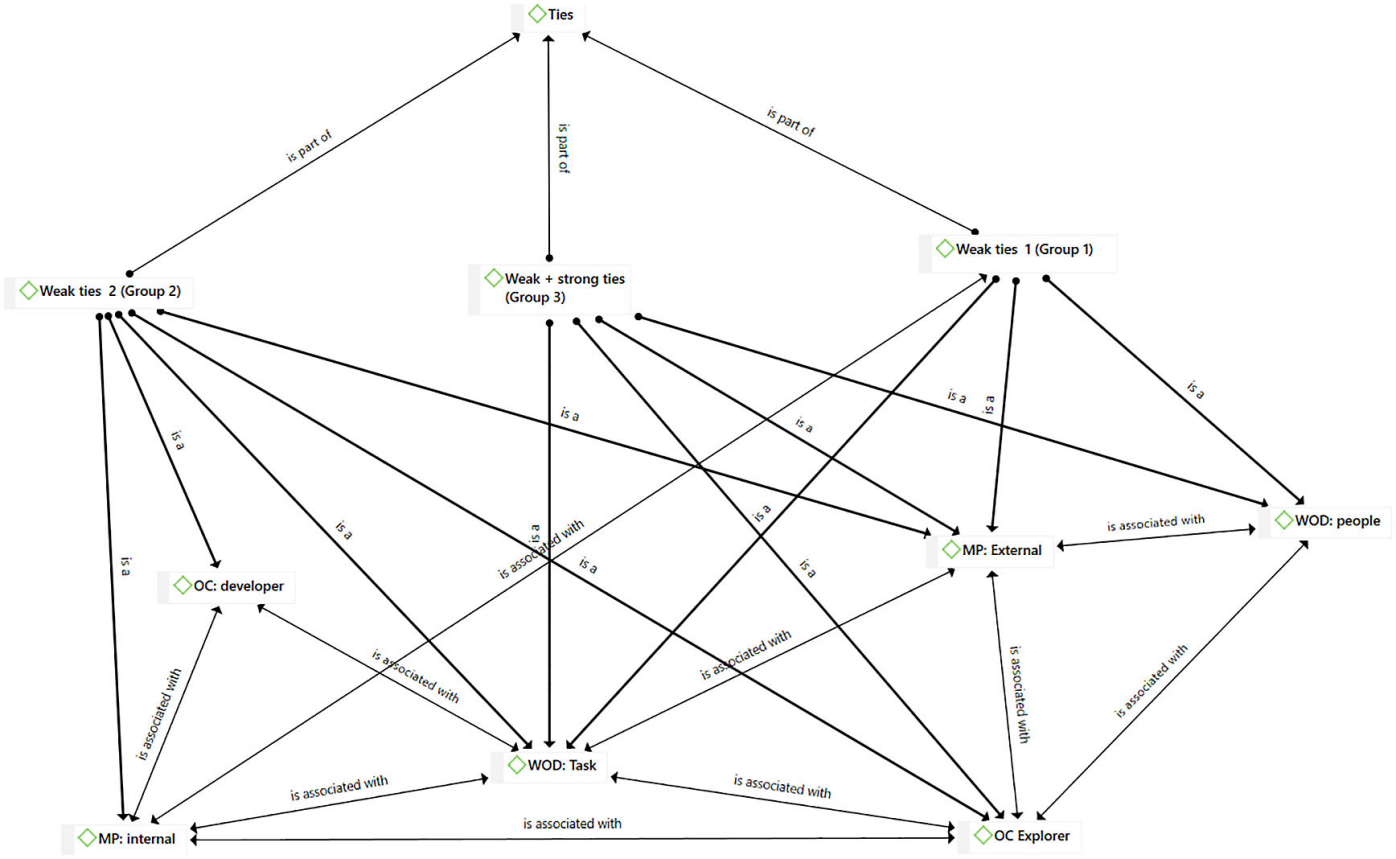
The network analysis from ATLAS.ti.

Figure 1 displays the network analysis based on the themes extracted from Facebook discussions.

**Figure 2.**
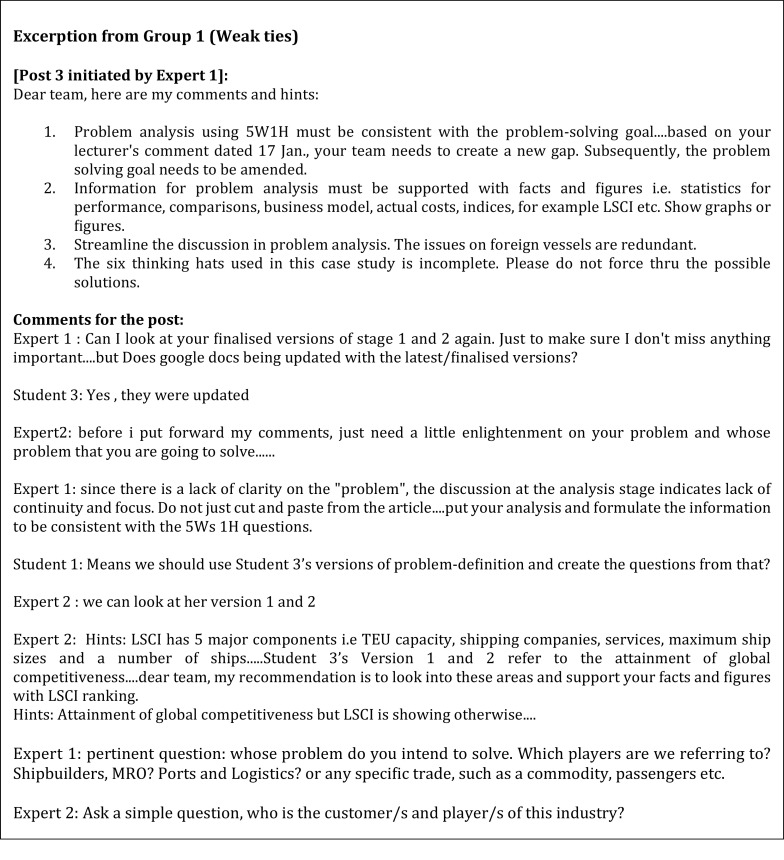
The weak tie from Group 1.

A closer examination of each group disclosed some differences in the problem-solving styles that the experts used. The weak tie experts in Group 1 ( Figure 2) displayed a few combinations of problem-solving styles. The experts applied a more accommodating approach and showed a sense of belongingness by using phrases such as “dear team” and “keep moving team”. Selby *et al.* ^15^ described this as the people preference style where this approach is seen as an effort to maintain harmony in the group. The experts also respected the students’ own pace of processing information. Besides that, they required time to digest and internalise the meaning of the information presented to them by the students before responding. This resembles internal style. However, the experts also tended to use the explorer style and expected students to contribute some ideas after the experts presented their points or when the experts would like to comprehend an issue.

**Figure 3.**
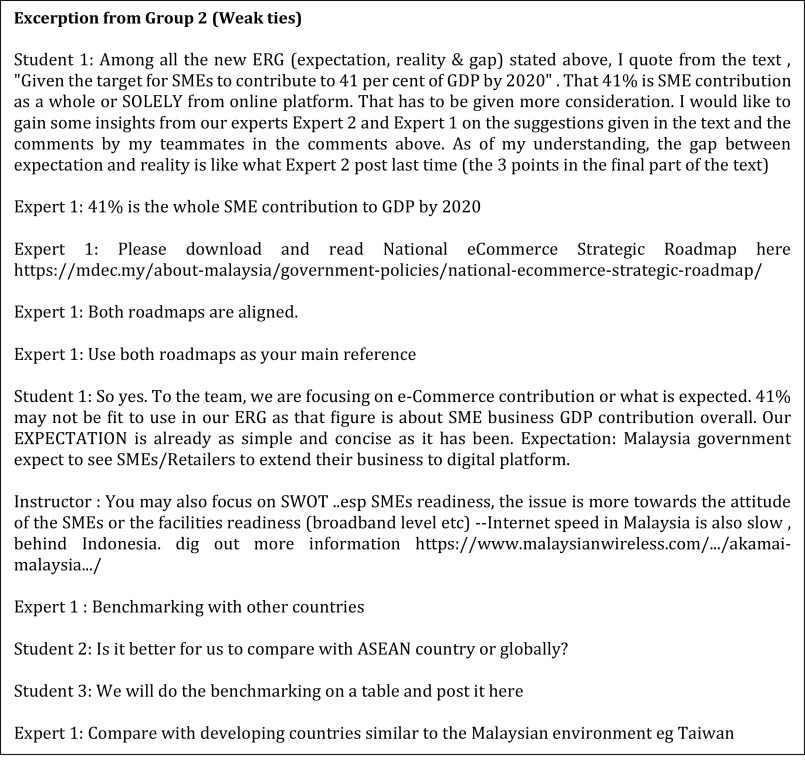
The weak ties from Group 2.

In contrast, the business experts from Group 2 (weak ties 2) ( Figure 3) adjusted their reasoning based on the information the students presented to them first. The experts preferred the students to explore all possible options and present the latest information before guiding the students based on the materials presented. This sort of arrangement falls under the explorer style. However, the experts mostly engaged with external style or explorer style only after the students probed them questions. Eventually, once they were able to decipher the information, they gradually exhibited a more task preference style, where the tone of the discussion was more towards task accomplishment and tended to be free from emotion.

**Figure 4.**
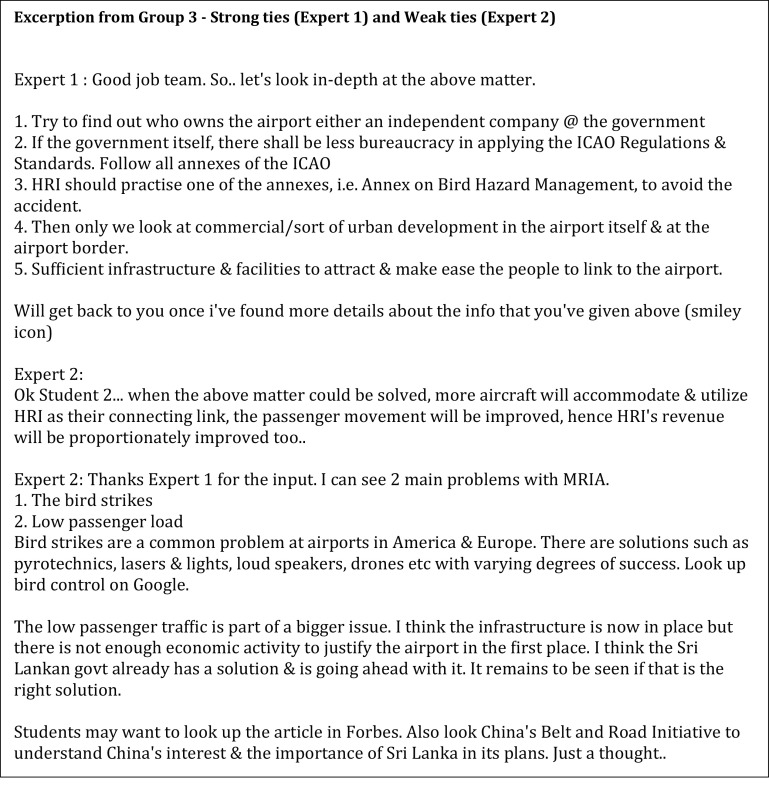
The strong and weak ties from Group 3.

Lastly, Group 3 which used a combination of both weak and strong ties ( Figure 4) showed mixed findings. The strong tie expert (Expert 1 from strong tie) was sensitive to the participants’ feelings and ended her comments with remarks such as “Otherwise, good job all”. This style is categorised as the people preference. The strong tie expert also demonstrated more persistence and patience in scaffolding the students by presenting the developer style. She directed the students beginning with a basic idea and gradually developed the ideas as the students were progressing by making statements such as “I think it would be a good idea if … .”. This characteristic is similar to the style of experts in Group 1. In contrast, the weak tie expert (Industry Expert 2) mostly displayed a task preference style and the explorer style after receiving information from the students. However, the expert from the weak tie in Group 3 deliberated more insights compared to weak ties’ experts in Group 2.

These sorts of problem-solving styles of the experts are correlated with their working experiences. Experts from Group 2 (with 10-15 years of work experience) were flexible in dealing with new information in thinking and reasoning with the students mostly after more information was supplemented by the students. Nevertheless, they provided information lacking in detail to be applied in the context of the problem. In contrast, the business experts in Groups 1 and 3 (with more than 30 years of work experience) demonstrated and shared validated business solutions by occasionally sharing how the presented information was linked to their past experiences. The explanations given by the experts in Groups 1 and 3 were also seen as more insightful compared to the guidance provided by experts in Group 2, for instance, they guided the students to the correct discussion paths by ensuring the students supply up to date information and present information in a logical manner. 

## Discussion and conclusion

According to Bilalić, McLeod, and Gobet, ^18^ the greater the degree of expertise, the more flexible the experts are in responding to new information. Experts from Group 1 and 3 were seen flexible in switching between developer and explorer style, possibly due to vast experiential knowledge, rendering them capable of deciphering information from different perspectives. The experts mostly used developer style in a combination of internal style when they wanted to check their understanding. For instance, the experts requested, “Can I look at your finalised versions of stage 1 and 2 again. Just to make sure I don’t miss anything important” or “I have vetted the case study”, which could be inferred that the experts needed some time to appraise the information that the students gave and tried to put the “pieces of the information together” in a way students could understand and use it. Additionally, it was observed that the experts from both groups sometimes expressed experience-based statements such as “I am very familiar with the local shipping industry. I hope this information can provide some clues” and “In aviation, we called it the bird strike hazards”. Selby *et al.*, ^15^ described, this as one of the characteristics of developer styles whereby the problem-solvers framed the discussions based on their present work experience and formulate practical working solutions that can be assimilated into the current reality.

However, they switched to explorer styles when they noticed students started to deviate from the discussion goals. Holton and Clarke ^28^ noted, experts with complete conceptual knowledge can guide students greatly such as providing forewarning against lacked in progression or mistakes because they know this knowledge exists. In this study, the experts alerted the students by urging cautions such as “your lecturer has mentioned Liner Shipping Connectivity Index. Get hold of it and understand it carefully. Use it in the Malaysian context” or asked probing questions like “Whose problem do you intend to solve? Which players are we referring to? Shipbuilders, maintenance, repair, and operations (MRO) operators? Ports and Logistics? or any specific trade, such as a commodity, passengers, etc”. By virtue of having subject-knowledge expertise allowing them to guide students more flexibly and better at posing questions to accommodate critical learning points. ^28^ Nevertheless, the experts used more technical terms and jargon which necessitated the students to ask a second party to provide the meaning-making for them. Occasionally, the students were observed needing to rely on the other expert or instructor for the meaning-making process (to put the meaning in a context understandable to the students). This is supported by Ryberg ^22^ who claimed that placing students in different degrees of ties sometimes require different participants like the instructor to provide the interpretation of meaning.

In contrast, experts from Group 2 had different problem-solving styles with the students. Instead of offering the information asked by the students straightaway, the experts from Group 2 often asked the students to search for the materials first, and later worked on the materials together with the students. This reflects the internal style of processing information. This was possibly done to avoid offering inaccurate advice and to verify the information before formulating relevant strategies to scaffold the students. However, Boshuizen, Grubber, and Strasser ^29^ asserted, intermediate experts usually lacked cognitive capacity when solving problems and may acquire more concepts to better connect existing knowledge networks. In other words, they may have basic concept knowledge but may still require assistance to make the basic knowledge more complete before it could be transcendent into a specific application context. ^30^ Possibly, the experts lacked situated knowledge, knowledge within the context of an individual environment or where one is currently located, ^31^ that allowing them to quickly adjust the relevant information embedded in the current context of the problem.

This might explain why experts in Group 2 mostly provided policy papers rather than offering specific real-life business evidence that the students could use as a reference. As a result, the experts tended to share information in a broader sense, for example, the experts recommended, “Benchmarking with other countries”, “Do the SWOT analysis on each component of digitization/e-commerce e.g., platform, payment, logistics and fulfillment, small-medium enterprises readiness and others”. Whereby, Group 1 and 3, the experts usually shared information by detailing the sources and the connections of the sources with the specific needs of the problem, for instance, “Try to find out who owns the airport either an independent company or the government. If the government itself, there shall be less bureaucracy in applying the International Civil Aviation Organization (ICAO) Regulations & Standards. Follow all annexes of the ICAO”.

One common similarity that all of these experts demonstrated was they needed initial information from students before they could fine-tune their scaffolding strategy. This could be implied as initiating a pre-scaffolding strategy. Although experts have more experiential knowledge than novices, experts sometimes need to rely on students and instructor to scaffold their initial understanding. This is consistent with social constructivism viewpoints on the need for knowledge to be co-constructed with others in a dialectical process through problem-solving experiences, to guide thinking and meaning-making towards a more complete understanding. ^32^ Business problems are usually more intricate to be understood as the problems contain multidisciplinary data sources and sometimes lacked business evidence-based practices. ^29^

Since each business problem is unique, experts may not be able to find complete answers and may rely on their current expertise or other people’s assistance that could be seen as an effort to obtain reciprocal scaffolding from other team members. Each member in the group at different points may have different expertise, hence, they may reciprocate their scaffolding to help others. ^28^ The students in this study may have more knowledge about the initial background of the problem, which prompted the experts to exhibit explorer style in combination with external style to gauge the initial information from the students. Once the relevant information was accumulated, they needed time to comprehend the issues by displaying developer and internal styles before channeling to task preference style and eventually exchanging with the students on task-related information. Occasionally, the students and the experts interchangeably played the experts’ roles, but the business experts displayed firmer scaffolding roles due to the wider conceptual and experiential knowledge than the students.

In this study, we concluded, all experts displayed orientation to change (explorer vs. developer) and the manner in processing information (external vs. internal) but the degree of its usages depends on the working experiences or situated knowledge that the experts had. However, one noticeable finding was the use of task and people preference style correlates with the past relationship that the students and the experts had. Experts from Group 1 and 3 had a past working relationship with one of the students in each group during internship placement led the business experts to display a more empathic attitude towards the students’ learning needs. In contrast, the business experts from Group 2 had no prior relationship with the students, thus their preference for using more task-oriented problem-solving styles that seemed lacking in granting supports such as providing encouragement and showing efforts to maintain group harmony. Although weak ties lack emotional closeness and reciprocal actions, ^33^ the finding of this study showed the ties with prior relationships help students alleviate available hurdles, which are in line with Castañeda and Selwyn ^34^ connotation of humanising the technology adoption and learning process itself. This study also verified that scholars should not equate all weak tie experts share similar problem-solving styles. It is postulated that how the students knew the business experts matters. Nonetheless, despite their different styles, the inclusion of the experts in the problem-solving discussions still accelerated the students’ learning, in tandem with previous studies that acknowledged business experts’ inclusion in PBL enhances students’ learning experience. ^35^^,^^36^

## Conclusion

This study contributes towards our understanding of the roles of problem-solving styles and the strength of ties in problem-solving activities on Facebook. The use of networked learning in PBL depends on individualised networking and social collaboration that encourage content generation in problem-solving. ^22^ It can be concluded from the findings that not all experts from the weak ties have similar problem-solving styles. Factors such as the experts’ work experience and how the weak ties were developed played a major role in determining the experts’ problem-solving styles, which indirectly influenced their thinking and reasoning strategies with the students.

The experts, regardless of whether they were from weak or strong ties, still benefited the students in expediting their problem-solving tasks. Thus, inviting business experts to participate in formal learning on social media by utilising the strong and weak ties the students have should be encouraged as each expert has unique expertise to offer, especially in helping the students see the different sides of complex information that are essential to prepare for their future career.

### Limitations

The use of non-probability sampling involving two experts in each of the three groups in one degree-level management course limits the generalisability of the findings to other courses. Hence, the study’s findings should be evaluated with caution and may only be applied to similar studies, for example, those that examine Facebook use for PBL in management courses.

## Data availability

### Underlying data

Figshare: Facebook Discussion with Business Experts (transcribed), https://doi.org/10.6084/m9.figshare.16811542.v2. ^26^

This project contains the following underlying data:

• Full transcribed data:
  • Datafile 1: Transcribed conversation of Group 1
  • Datafile 2: Transcribed conversation of Group 2
  • Datafile 3: Transcribed conversation of Group 3

• Data coding:
  • Datafile 4: Orientation to change (OC) developer style
  • Datafile 5: Orientation to change (OC) explorer style
  • Datafile 6: Manner of processing (MP) external style
  • Datafile 7: Manner of processing (MP) internal style
  • Datafile 8: Way of deciding (WOD) task preference style
  • Datafile 9: Way of deciding (WOD) people preference style

Data are available under the terms of the Creative Commons Attribution 4.0 International license (CC-BY 4.0).
