# Supplementary material for: Weak and strong ties and its connection to experts' problem-solving styles in scaffolding students' PBL activities on social media
Source: F1000Res. 2021 Dec 16;10:1076. Originally published 2021 Oct 25. [Version 2] doi: 10.12688/f1000research.73210.2 (PMC8729031; doi:10.12688/f1000research.73210.2)
Supplement: Peer review history

# Peer review: Reviewer response for version 2

**DOI:** 10.5256/f1000research.80117.r115451

**Published:** 2021-12-24

**Authors:** Els Boshuizen

**Affiliations:** 1 Faculty of Educational Sciences, Open University, Heerlen, The Netherlands

I am very pleased about the way the author revised the article. It is very well readable now, and makes an interesting contribution to what we know about how ways of expert scaffolding of student learning processes.

Is the work clearly and accurately presented and does it cite the current literature?

Partly

If applicable, is the statistical analysis and its interpretation appropriate?

Not applicable

Are all the source data underlying the results available to ensure full reproducibility?

Yes

Is the study design appropriate and is the work technically sound?

Partly

Are the conclusions drawn adequately supported by the results?

Partly

Are sufficient details of methods and analysis provided to allow replication by others?

No

Reviewer Expertise:

Expertise development; expert vs novice knowledge and knowledge structure; expert vs novice reasoning.

I confirm that I have read this submission and believe that I have an appropriate level of expertise to confirm that it is of an acceptable scientific standard.

# Peer review: Reviewer response for version 2

**DOI:** 10.5256/f1000research.80117.r115449

**Published:** 2021-12-23

**Authors:** Fauziah Sulaiman

**Affiliations:** 1 Faculty of Science and Natural Resources, Universiti Malaysia Sabah, Kota Kinabalu, Malaysia

I have read the revised article and it seems all corrections were done accordingly. Thus, from my side, I don't have any issue in accepting the article for further process.

Is the work clearly and accurately presented and does it cite the current literature?

Yes

If applicable, is the statistical analysis and its interpretation appropriate?

Partly

Are all the source data underlying the results available to ensure full reproducibility?

Yes

Is the study design appropriate and is the work technically sound?

Yes

Are the conclusions drawn adequately supported by the results?

Yes

Are sufficient details of methods and analysis provided to allow replication by others?

Partly

Reviewer Expertise:

NA

I confirm that I have read this submission and believe that I have an appropriate level of expertise to confirm that it is of an acceptable scientific standard.

# Peer review: Reviewer response for version 1

**DOI:** 10.5256/f1000research.76848.r97802

**Published:** 2021-11-23

**Authors:** Lillian Buus

**Affiliations:** 1 VIA University College, Aarhus, Denmark

The article works with weak and strong ties in relation to PBL group-work inviting in experts to scaffold students learning. 

 This is an interesting and relevant research, although I miss a clear theoretical approach, as it seems the theory is not following through the article. The authors present in the introduction 'connectivism' but do not refer to this or their arguments in the introduction or the discussion. I also wish to stress that the first two references are placed wrong and that Siemens is missing an s (just to have some formats in place). 

 What I would suggest to the authors, is to work a little more with the structure of the article as well. I miss a clear theoretical part, that also can be part of the argumentation in the discussion. So, choose only some of the theoretical approaches brought in, and go more in depth with these arguments. 

 Another part I would like the authors to consider is the figures. It might be good to structure the 'Excerption from the groups' after each other, as it becomes confusing to read the analysis 'in between' figures. I also miss the arguments for the statement: "The explanations given by the experts in group 1 and 3 were also seen as more insightful compared to the guidance provided by experts in Group 2" - I miss the "Why" and "How" arguments on this. 

 I also would like the authors to elaborate more on the analysis and how they have conducted that e.g. with examples of parts that identified the expert’s roles. Also, for other being able to in details to replicate the research. Transparency is important, and there is a minor lack in this part. 

 A part I also think is missing in the methods is the ethical consideration on using SoMe like Facebook for this. Not that it is wrong but I believe that there is some issue combined with this. What if an expert didn't have Facebook or would use this media for professional work issues? What about the right for information on Facebook? Other ethical issues could also be stressed. Just a notion on this could be expected as well when talking ethical issues. 

 It was positive to read that the authors took into consideration the sampling for the research, which is good. I think the research is interesting and I would like the authors to go more into this to gain more insights for this. 

 Good work so far and just work on the combination of theoretical arguments and analysis/conclusion will give your article more strength.

Is the work clearly and accurately presented and does it cite the current literature?

Partly

If applicable, is the statistical analysis and its interpretation appropriate?

Not applicable

Are all the source data underlying the results available to ensure full reproducibility?

Yes

Is the study design appropriate and is the work technically sound?

Partly

Are the conclusions drawn adequately supported by the results?

Partly

Are sufficient details of methods and analysis provided to allow replication by others?

Partly

Reviewer Expertise:

Expertice in Problem-Based learning and education, knowledge about strong and week ties, from novice to expert (Dreyfuss and Dreyfuss), Teaching with digital technologies and social media use,

I confirm that I have read this submission and believe that I have an appropriate level of expertise to confirm that it is of an acceptable scientific standard, however I have significant reservations, as outlined above.

# Peer review: Reviewer response for version 1

**DOI:** 10.5256/f1000research.76848.r97803

**Published:** 2021-11-09

**Authors:** Mohd Nazir Md Zabit

**Affiliations:** 1 Department of Educational Studies, Universiti Pendidikan Sultan Idris, Tanjong Malim, Malaysia

This paper aims to explore the problem-solving styles that the experts portrayed on Facebook based on their ties with the students.

 Overall, the methodology suits the research requirements. It has been clearly explained and easily understood by readers. The only concern is how the authors arrange and monitored the three closed Facebook groups with 12 final year management students, six business experts, and one instructor as the participants. Need a little bit of explanation.

 Overall, the manuscript has been written and prepared in a moderate writing style. The work is acceptable based on the suggestions given and should also be based on other assessors’ comments.

Is the work clearly and accurately presented and does it cite the current literature?

Yes

If applicable, is the statistical analysis and its interpretation appropriate?

Not applicable

Are all the source data underlying the results available to ensure full reproducibility?

Yes

Is the study design appropriate and is the work technically sound?

Partly

Are the conclusions drawn adequately supported by the results?

Yes

Are sufficient details of methods and analysis provided to allow replication by others?

Partly

Reviewer Expertise:

Business education; teacher pedagogy; thinking skills

I confirm that I have read this submission and believe that I have an appropriate level of expertise to confirm that it is of an acceptable scientific standard, however I have significant reservations, as outlined above.

# Peer review: Reviewer response for version 1

**DOI:** 10.5256/f1000research.76848.r97807

**Published:** 2021-11-08

**Authors:** Fauziah Sulaiman

**Affiliations:** 1 Faculty of Science and Natural Resources, Universiti Malaysia Sabah, Kota Kinabalu, Malaysia

Overall the article is well written and easy to follow. It is about the use of PBL in the Facebook platform to students' engagement in real-life activities by involving the business expert as the booster for them to really immersed in the issue they are facing. However, the weak and strong ties in this research are lacking with basic info, for example, why does the student need to choose by themself the business expert? Why not the researcher choose the business expert? Therefore the weak, strong, and weak+strong group setting maybe can be arranged. Having these totally different group settings can lead maybe different impactful outputs. Just a thought anyway. 

 So these are my comments on the statement that I commented as partly:)

 Other than that, all looks adequate.

 A few additional comments on the manuscript can be found here.

Is the work clearly and accurately presented and does it cite the current literature?

Yes

If applicable, is the statistical analysis and its interpretation appropriate?

Partly

Are all the source data underlying the results available to ensure full reproducibility?

Yes

Is the study design appropriate and is the work technically sound?

Yes

Are the conclusions drawn adequately supported by the results?

Yes

Are sufficient details of methods and analysis provided to allow replication by others?

Partly

Reviewer Expertise:

Education: Physics/Science Education, Educational Technology, Higher Education, in-depth in Problem Based Learning Model. Physics: Non-Destructive Testing (Eddy-Current Testing Technique)

I confirm that I have read this submission and believe that I have an appropriate level of expertise to confirm that it is of an acceptable scientific standard, however I have significant reservations, as outlined above.

# Peer review: Reviewer response for version 1

**DOI:** 10.5256/f1000research.76848.r97804

**Published:** 2021-11-02

**Authors:** Els Boshuizen

**Affiliations:** 1 Faculty of Educational Sciences, Open University, Heerlen, The Netherlands

This article investigates how external experts (that is, not connected to the university) with weak or strong ties to the students can support them through social media in their problem-based learning activities.

 It is investigated in three PBL groups of four students each, who worked on divergent topics, and who invited two domain experts per group. The Facebook platform was used for communication and cooperation as it provides ample opportunities for data collection.

 A couple of theoretical foci were applied: strong vs weak ties; PLE as a means to receive on-line scaffolding; expertise seen as fluid vs crystallised intelligence; aspects of expert-novice interaction; Selby's problem solving styles; system1 vs system2 thinking. One could say that this is an innovative combination of theoretical approaches, however - in my view - it blurs the real innovation in this study, that is, the participation of outsider-experts in student support through many different expert actions: providing information and information sources, pointing out gaps, asking questions, etc. combined (or not) with motivating actions. Also the role of the instructor as a go-between students and experts is an interesting addition. And I agree with the authors that the participation of these experts was successful.

 Having said that I list a couple of issues that need clarification:

1. The authors should make clear why they introduced the theory of fluid and crystallised intelligence to describe expert reasoning and knowledge use in combination with interaction with novices. Using this theory introduces connotations that might not be intended. Especially in a short article like the present one, it is better to be sparsimonious with concepts used. Very much research has been done on the issue expert-novice communication in PBL including the question whether intermediates might fulfil a better role (for instance by Schmidt and Moust) that does not use these concepts.
2. Furthermore, the authors should better document how the use of fluid vs crystallise intelligence was coded. And in the Results section it is good practice to provide proof using quotes. The excerpts given provide interesting reading material but they do not show how the researchers coded this.
3. The issue of strong vs weak ties is very interesting. I wonder whether the way expert invitation was carried out was sensitive to tie strength. I assume that available expertise was the decisive factor, not kind of tie. It is, of course, interesting whether kind of tie affects communication.
4. The issues raised by Bilalic et al. and by Kahneman resonate in different ways with fluid and crystallised intelligence. They are very interesting but by introducing these authors it is more difficult to bring the message across.
5. Small issue p7: According to Bilalic, McLeod, and Gobet .. McLeod is missing.
6. As the journal's focus is on fast publication, I would prioritise the comments on Method and Results.

Is the work clearly and accurately presented and does it cite the current literature?

Partly

If applicable, is the statistical analysis and its interpretation appropriate?

Not applicable

Are all the source data underlying the results available to ensure full reproducibility?

Yes

Is the study design appropriate and is the work technically sound?

Partly

Are the conclusions drawn adequately supported by the results?

Partly

Are sufficient details of methods and analysis provided to allow replication by others?

No

Reviewer Expertise:

Expertise development; expert vs novice knowledge and knowledge structure; expert vs novice reasoning.

I confirm that I have read this submission and believe that I have an appropriate level of expertise to confirm that it is of an acceptable scientific standard, however I have significant reservations, as outlined above.

# Response

**Published:** 2021-11-02

**Authors:** Aznur Hajar Abdullah

**Affiliations:** Multimedia University , Malaysia

Dear Prof Els Boshuizen,

 We are greatly appreciative of the insightful comments and helpful suggestions that you have provided.

 Some of the issues are somewhat similar in term of the responses, so we encapsulated issue 1 and 3, in the last comments (comment no# 5).

 The following are our response on the five issues that you have highlighted:

1. Thank you for proposing Schmidt and Moust’s paper, we had read the paper with great interest. Schmidt and Moust studied about what makes a tutor effective and we found some similarities in the line of argument, that also emphasizes the personal qualities of the tutor . In essence this study proposed, do not presume experts from similar ties would offer similar degree of scaffolding as their degree of scaffolding corresponds with the level of crystallised intelligence that they have .
  We have commented more detailed in comment #5 below ).
* In the revised version later, we will incorporate more clearly on why t the theory of fluid and crystallised intelligence was used.
3. In the original submission the codes were attached, but they were coded directly from Facebook communication ( all Facebook communications were the snapshots from Facebook and coded in ATLAS.ti). Despite the identities of all participants were cancelled and made anonymous , for copyright purposes, the F1000 reviewer recommended to transcribe the conversations as they cannot be published with any element associated to Facebook.
  *In the revised version, we will incorporate again the coding.
4. To some extent we believe the ties matter as strong tie and weak tie with more working experiences who showed differing in levels of intelligence (fluid vs strong intelligence) - they were more accommodative to students' learning needs. Explaining about the tie per se seems incomplete to explain why not all weak tie experts behave in similar manner when scaffolding the students. Experts from Group 2 showed different scaffolding approach from experts from Group 1 and Group 3 . We have commented more detailed in comment #5 below ).
5. Rather than looking at Bilalic and Gobet resonate differently from Kahneman’s, we opine that the arguments from these authors complement the findings of this study. Bilalic and Gobet resonate the flexibility of the experts is in accordance with the degree of fluid and crystallised intelligence that the experts have. This study showed that, experts with lesser working experiences tended to seek  “help” or to rely on information provided by the students before they could fully utilised System 2 thinking to scaffold the students to deal with ill-structured problems. Although novices have to go through certain stages before they could pick up and put pieces of information into a meaningful context, your comments made us realised, the experts with less crystalised intelligence need to put pieces of information too. Interestingly, we view this as a pre-scaffolding preparation that experts from Group 2 in this study exhibited before they could devise an appropriate assistance to students. Your comment has enlightened us to propose future research to investigate the reciprocal role that students could play in scaffolding the experts’ thinking.

In the revised version we will improve the clarity of the sentence to capture the above comments in the revised version. If you agree with the above comments, please let us know.

 Thank you again for your time in reviewing this manuscript.
